# Analysis and Psychoeducational Implications of the Behavior Factor During the COVID-19 Emergency

**DOI:** 10.3389/fpsyg.2021.613881

**Published:** 2021-02-16

**Authors:** Jesús de la Fuente, Douglass F. Kauffman, Michael S. Dempsy, Yashu Kauffman

**Affiliations:** ^1^School of Education and Psychology, University of Navarra, Pamplona, Spain; ^2^School of Psychology, University of Navarra, Pamplona, Spain; ^3^Medical University of the Americas–Nevis, Devens, MA, United States; ^4^Bost University, Lashkar Gah, Afghanistan; ^5^William James College, Newton, MA, United States

**Keywords:** COVID-19, psychology, educational psychology, self-regulated learning vs. externally-regulated learning theory, implications for intervention

## Abstract

This theoretical analysis seeks to contribute to three objectives within the context of the proposed *Frontiers* Research Topic: (1) delimit two levels of analysis in the present pandemic situation: medicine-epidemiology and behavioral psychology, still under-addressed. While medicine has its essential role on the biological side, psychology has a comparable role on the behavioral side. (2) Analyze the importance of behavioral-educational factors in the pandemic situation, using a precise theoretical model from educational psychology for this analysis. (3) Propose preventive, psychoeducational intervention strategies based on the previous analyses.

## Introduction

When the COVID-19 pandemic appeared in early 2020, every effort was made in the scientific and professional world to understand its impact, know how the virus functions, and develop strategies for prevention and eradication (Brooks et al., 2020; Chacón-Fuertes et al., 2020; Leung et al., 2020). Thus, an enormous amount of basic and applied research has been dedicated to this purpose (Alessandri et al., 2020; Castelnuovo et al., 2020; WHO Regional Office for Europe, 2020). Given the characteristics of this health problem, epidemiological science – as a scientific branch of medicine – has played a leading role in the profusion of data, design of preventive measures and population monitoring, as well as in establishing measures to contain the disease. Considerable research also addresses the problem of fighting the disease in a therapeutic sense, from the biological and biomedical standpoint (Huremovic, 2019).

From a psychological viewpoint, research has largely been aimed at assessing the impact on persons’ mental, emotional and behavioral health from the periods of confinement that a large part of the population has been subjected to (Lippold et al., 2020; Spalleta et al., 2020). The effects of confinement on mental health have been analyzed by a number of authors (Taylor, 2019a,b,c; Sokolowska et al., 2020). Parallel analyses have also been carried out from the viewpoint of *cultural and socio-communitarian psychology*, as well as from positive psychology (Whillans et al., 2020) and from the approach of the mass media (Arriaga et al., 2020).

However, these studies do not exhaust the analysis of the pandemic phenomenon and its many components, given that there are other perspectives. The *Educational Psychology perspective* is relevant and specific because it focuses on the impact that this new situation is having on the developmental and learning processes of the persons involved, not to mention the preventive behavioral change that is needed to adaptively face this unprecedented situation (de la Fuente et al., 2020b). Understanding this reality is especially important in the different educational environments, such as the family, the school, the socio-educational context, and the new technological contexts of nonformal education (Li and Xie, 2020). It is also necessary to analyze the different stages of education (Cao et al., 2020; Wilke et al., 2020). The present research topic addresses this object of study, from a psychoeducational perspective (de la Fuente et al., 2020b).

In response, this study seeks to contribute to three objectives within the context of the *Frontiers Research Topic*: (1) delimit two levels of analysis in the current situation: the biological level and the – still under-addressed – behavioral level. While medicine has its essential role in the biological side, psychology has a comparable role in the behavioral side. (2) Analyze the importance of behavioral-educational factors in the pandemic situation, using a precise theoretical model of analysis. (3) Propose preventive, psychoeducational intervention strategies based on the previous analyses.

## On Human Hardware and Software: the Roles of Medicine and Psychology in Health

When students ask what the difference is between the object and scope of Medicine vs. Psychology, the computer offers a graphic metaphor (de la Fuente, 2020a). This metaphor makes it easier to understand that human beings can be categorized into two levels for analysis: their “hardware” and “software.” The *hardware level of analysis* refers to the structural and functional aspects of the human being’s physical equipment (biological, physiological, biochemical, neurological, and hereditary), with which we come into the world. Included are all the body’s biological systems, structural and functional, that allow it to be a living organism in its environment. It is then the structural and functional equivalent of the computer’s physical equipment that allows it to function as such (cables, connections, and microchips). The *software level of analysis* refers to structural and functional aspects that are psychological in nature, allowing the human being to acquire the specific learning programs for behavioral adaptation to his or her environment, through education. Consequently, this level is comparable to programs installed on a computer, allowing it to execute tasks and productive actions: data analysis, word-processing, graphic design, machine learning, big data, and so on.

Anyone who understands computers knows that optimal hardware (memory, motherboard, and processing speed) and adequate software (installed programs) are both required. It is possible for good hardware to not be accompanied by good software. In other words, optimal physical equipment will not achieve high standards of performance if not accompanied by optimal programming, and vice versa. In the sphere of human development, this concept is classically referred to as nature (genetic) vs. nurture (learning/environment). For many years, the controversy on genetics (hardware) vs. environment (software) filled endless numbers of studies and research positionings as to the relative weight of the two factors. We know today, and it is obvious to all, that the two factors combine to determine the final human product.

There are two clear distinctions, however, where the computer metaphor falls short. On one hand is the material with which computers and people are composed. While the computer contains hardware built with physical components (silicon, etc.), the human being has biological or biochemical hardware. On the other hand, while computer hardware and software do not modify each other when interacting (at least, in the classical design), such modification does take place in the human being.

Concerning the first issue, *biological hardware* can explain a significant part of behavior, but not all. Genetics, physiology and neurology are essential to explaining the biological basis of human behavior. This is the sphere of medicine (biological, physiological, biochemical and neurological science, i.e., human hardware). However, common knowledge and formal science both offer evidence that learning and experiences also play an important role in accounting for human behavior. This is the sphere of *psychology* (the science of behavior or of human software). Put differently, human beings and their health are the object of study of both sciences. In the former, biological explanatory models are created (biological hardware models), while in the latter, models are based on behavior (behavioral software models). As with computers, it is easily understood that neither of the two parts, on its own, can fully explain effective functioning. The historical error lay in trying to explain human functioning solely on the basis of the hardware. In other words, not enough weight was given to behavior in explaining health issues and level of functioning. This first idea has a central implication in the health sphere: the factors that determine health include both hardware (biological equipment) and software (the programming that results from behavior). Physical (biological) health and psychological (behavioral) health are equally important.

As for our second distinction, human beings are unlike computers in that the two levels of hardware and software are interconnected and modify each other. As noted above, the software in a computer – at least, on classic computers – does not usually modify how the hardware functions. The fact that this does occur in the human being must be analyzed if we wish to explain deficiencies or optimize functioning. In the human being, the biological interaction between software and hardware is not mechanical and it produces cross effects, unlike a computer. In other words, implantation of a certain “software program” (psychological experiences or human learning) ends up affecting and modifying the initial hardware (biological equipment). While many classical diseases are obviously problems specific to the biological level (malfunctioning of biological hardware, such as organs), epidemiological studies show that many other diseases are produced by poor acquired habits, consolidated over time (inadequate “software,” such as smoking or alcohol abuse). Such habits finally have negative repercussions on the organs or systems involved, and disease appears at the biological level (lung cancer or cirrhosis). Therein lies the value of learning experiences (“software”) in health problems.

For this reason, experts in human hardware (biologists and medical doctors, in general) analyze the structure and functioning of the physical equipment, studying at great length how to ensure good biological functioning. Neurologists, for example, study and analyze the biological, structural, and functional equipment of the brain; cardiologists, the structure and function of the heart and the circulatory system. By contrast, experts in human software (health and clinical psychologists, in particular) analyze the structure and functioning of common learning programs, that is, habits, and the behavioral and emotional factors involved in the health issue at hand, using models constructed from recent evidence to explain health and disease.

This differentiation is key to understanding these two complementary lines of work in the sphere of health. While *Medicine*, as a biological science, focuses on the study of physical or biological factors of health (human hardware factors), *Psychology*, as a behavioral science, studies the behavioral factors of health (human software factors). While it is true that one branch of Medicine, Psychiatry, concerns itself with Mental Health, we must not forget that its models of assessment and intervention are preferably medical, that is, pharmacological, neurological or psychotherapeutic. Perhaps in this way we can understand that psychologists are very much needed in common health issues (general healthcare psychologists and qualified healthcare workers). Examples include psychological monitoring of patients with cancer, improving treatment adherence in chronic disease, designing programs to optimize emotional and behavioral health in senior citizens, determining the psychological and behavioral impact of pharmacological treatments, and so on. This is even more evident now with the COVID-19 pandemic.

Consider the evidence and today’s knowledge of how psychological programs produce beneficial effects in the emotional and behavioral adjustment of patients with cancer. Is it comprehensible in the 21st century that these treatments are not administered in Primary Care at hospitals, nor are they covered by medical insurance? Such treatment must be carried out outside the hospital context as an external activity. This is just one example that can be extrapolated to heart attack victims, chronic disease, smoking, post-traumatic stress, etc.

Empirical evidence supports the fact that preventive intervention in health care (software programs) – whether primary, secondary, tertiary or quaternary – forestalls many later hospitalizations (hardware issues). It is time that those responsible for healthcare management understand that hardware is not the only question, but also software to a large degree. We must, therefore, work toward whole-person intervention for improving health, both from the biological sphere and from the psychological. Much work remains to be done.

## The Role of Educational Psychology As an Applied Science, Within the Reality of the Covid-19 Epidemic

*Educational Psychology* is the branch of psychological science that analyzes educational problems using models and paradigmatic, scientific knowledge from psychology, applying it to education (de la Fuente et al., 2018). Working within each branch of knowledge leads to scientific and professional specialization, associated with a specific domain and level of study, whether micro-analysis (neuropsychology), molecular analysis (clinical psychology) or molar analysis (educational, social and health psychology; de la Fuente et al., 2019a). In today’s specific context, educational psychology as a discipline analyzes several problem areas:

1. How the COVID-19 experience is affecting the different aspects of *psychological development* at each age (Liu et al., 2020; Yoshikawa et al., 2020). If we adopt the approach of whole-person development (physical, socio-moral, motor, cognitive and linguistic), studying the impact in these different aspects should be of interest. Also of interest are peer relations mediated by technology, as well as the impact on pre-existing problems in this area, such as cyberbullying, sexting, and so on (Alemany-Arrebola et al., 2020). We analyze how this experience of stress produces effects in different stages of education, as well as how to address and promote behavioral health, based on models that have contributed empirical evidence.
2. How the COVID-19 experience is affecting processes of *academic and scholastic learning*. This involves analyzing the causes and effects of different forms of learning (Cachón-Zagalaz et al., 2020). Well-adjusted use of technology in the teaching-learning process, its effect, and associated emotionality are important. Researchers are also concerned with the experiences of adaptational stress in students, teachers, and parents within this scenario (Valadez et al., 2020). In complementary fashion, innovation in teaching processes, based on new technologies and methodologies, is of great interest (de la Fuente, 2020c).
3. How the COVID-19 experience is affecting *different educational contexts*. Effects within the family context are important when detecting problem areas and establishing guidelines for intervention. Also important is the effect on academic achievement and construction of scholastic and academic competencies, including the areas of special educational needs and attention to diversity (Alemany-Arrebola et al., 2020; Martarelli et al., 2020; Zaccoletti et al., 2020).
4. The *use and abuse of ICT* merits its own chapter, especially in developed societies, where the use of everything online has shot up exponentially during this period (Obrero-Gaitán et al., 2020).

## Epidemiological Vs. Behavioral Analysis For Prevention, During the Covid-19 Crisis

### Epidemiological Analysis for COVID-19 Prevention

From the epidemiological point of view, *health literacy* is the process of obtaining knowledge, motivation, and individual competencies to be able to understand and gain access to health-related information, to express one’s opinions, and to make decisions concerning the promotion and maintenance of one’s health. It is applicable in different contexts and settings throughout one’s life span (Lazcano-Ponce and Alpuche-Aranda, 2020). This conceptual perspective is very much needed in the present COVID-19 emergency, where the pandemic has produced devastating effects not only in health but also in the economic, political and social realms. These authors have established the elements that guide *public policies of prevention and control,* including:

1. *Epidemiological intelligence,* which includes not only the population surveillance strategy but, given the impossibility of identifying all positive cases, implementation of sentinel surveillance strategies and event-based surveillance (Proyecto Covid-19, 2020).
2. *Measures to mitigate the spread of the epidemic*, such as social distancing and hygiene, hand washing, quarantine, restriction of movement and use of face masks, among others. Clarity and certainty are critical elements for preserving trust and decreasing social concern. The following may be considered key community mitigation strategies:
  - Cancellation of private and public events.
  - School closures and suspension of religious services, musical and cultural events, etc. This element is key because these places are characterized by density of persons. For example, we find 3-5 m^2^ per child at school, compared to 18m^2^ per person at offices and 36-44 m^2^ per person, ideally, in homes.
  - Direct measures of social distancing and preventing close contact between members of a community.
  - Travel restrictions, including reduced flights, public transportation and restrictions on nonessential movement.
  - Voluntary quarantine of family members at home.
  - Restrictions on attending funeral services and minimizing exposure to bodily fluids.
  - Efficient communication from health authorities at the national and international levels, reporting true information and avoiding fake news, rumors and panic.
3. *Measures to stop the spread* when the number of cases is very high, such as more drastic measures of confinement at home or the *use of masks*. Here we can mention certain recommendations in the light of common errors in using a mask. Specifically:
  - Do not wear the mask under your nose.
  - Do not leave your chin exposed.
  - Do not wear the mask loosely, with open spaces on the sides.
  - Do not wear the mask so that it only covers the tip of your nose.
  - Do not place the mask under your chin, so that it rests around your neck.
  - Wear the mask so that it comes all the way up, near the bridge of the nose, and covers down below the chin.
  - Do your best to tighten the loops so that they fit to your face and do not leave open spaces.
  - Wash your hands before and after using a mask.
  - Hold the mask by the loops when putting it on and taking it off.
  - Avoid touching the front of the mask when taking it off.
  - For apartment dwellers, wear the mask when inside the building. Elevators and stairs can be highly contaminated areas.
  - Wash and dry cloth masks daily, and store them in a clean, dry place.
  - Avoid adopting a false sense of safety.
4. *Reinforcement of medical care capacity* in healthcare systems and increased ability to prevent transmission in healthcare services, including the use of diagnostic tests.

### Psychoeducational Behavioral Analysis for Prevention, in the COVID-19 Crisis

From the psychological or behavioral point of view, prevention in individuals and communities is a “problem of managing behavior change, based on a competency model, in order to respond to the new demands of the epidemiological context, contributing to non-contagion and stopping the spread of the virus, while ensuring a certain level of general health.” Let us carefully analyze the components of this definition:

1. *Management of behavioral change.* Producing behavioral change in the population is the most difficult task in a complex epidemiological context such as in COVID-19. For many years, the science of psychology has analyzed this object of study, conscious of the complexity of inducing or predisposing changes in human behavior. Scientific models of this phenomenon have been developed (Hagger et al., 2020). Educational Psychology, specifically, has analyzed the difficulties inherent in behavior changes in different educational contexts (de la Fuente, 2017). It is, therefore, neither scientific nor realistic to expect people to modify their behavior only because they are asked to do so, or by giving them information about the dangers of the virus. Such change – subject to complex behavioral laws – will only be possible if: (1) External conditions are adequately designed, predisposing change in the desired direction (context factors), and (2) the person has prior competencies for dealing with change (individual factors).
2. *Based on a Competency Model.* For behavioral change to be produced, the person must be competent, that is, must possess behaviors or prerequisite skills involved in such change. The characteristic competency model of educational psychology (Gagne et al., 1988; Gagne, 1997; de la Fuente, 2015) has established that a person is competent when three levels of behavior have been incorporated: KNOWING (behavioral knowledge and principles) + KNOW-HOW (behavioral skills and meta-skills) + MINDSET (behavioral attitudes, values and habits; Westera, 2001):
  *Knowing:* In the case that concerns us, this means having *adequate information* (causes, consequences, effects, mechanisms of transmission, types of masks, activities allowed, and harmful practices), as well as *adequate training* (personal principles of prevention, health and co-responsibility in view of the pandemic).
  *Know-how:* This refers to *instrumental skills* for managing the situation (from basic skills like putting on a mask, to complex social skills of interaction, stress management skills, and so on). Also included are *meta-skills,* essential and central to managing one’s skills, whether meta-emotional (coping strategies), meta-behavioral (self-regulation and problem solving), meta-motivational (resilience), and meta-learning skills (metacognitive strategies).
  *Mindset:* This essential level refers to one’s set of *attitudes, personal values* (character strengths), and *health habits.* It is especially important because it determines the level of motivation for adherence to behavioral change. Note that the subjective evaluation of a behavior’s importance is associated both with its inherent value and with the subjective perception of risk (Lohiniva et al., 2020). For this reason precisely, we must guard against messages like “it does not affect young people.” If we do not, it is highly likely that this population sector will feel invulnerable and become a very active source of contagion.
3. *The new demands of the new epidemiological context.* Everyone can observe that the new behavioral demands of the pandemic involve two types of complex behavioral readjustments:
  *A decrease in reinforcing behaviors, and behavioral inhibition of frustration* (resistance to frustration) that is inherent in losing the usual behavioral reinforcers (free time activities and fun, outdoor activities, and loss of economic resources for interaction with reinforcers). This modification falls under the behavioral paradigm of wanting and not being able to, or attraction-avoidance (Martarelli and Wolff, 2020).
  *An increase in restrictive and self-care behaviors,* as well as the sustained effort of continuing to perform them. This means dedicating effort and resources to new behaviors that were not carried out before (wearing a mask, keeping a safe distance, exponential increase in handwashing, new distribution of free time activities, etc.). This adjustment and behavioral modification falls under the paradigm of control-countercontrol (Martarelli et al., 2020).
4. *Contributing to stop the spread of the virus.* This involves an environmental redesign of the first order: limiting distance and social/interpersonal physical contact, as well as any human activity that promotes contagion. Given that many human reinforcers are of this type, a great effort is required in bearing with frustration and loss. This decreases the likelihood of positive emotional states and increases the likelihood of negative ones (de la Fuente, 2017).
5. *Ensuring a certain level of general health.* All this implies that a certain degree of psychological well-being must be attainable to manage the situation (Kanekar and Sharma, 2020). In other words, some amount of positive emotionality is needed to avoid falling into apathy or reactive depression, which are predisposed by such situations (Wolff et al., 2020). For this reason, resilience as a personal characteristic, attitude or first-order skill becomes essential, as do personal character strengths (Barzilay et al., 2020).

## Contributions From SRL Vs. Erl Theory To Psychoeducational, Behavioral Analysis and Prevention in the Covid-19 Crisis

### Contributions From SRL vs. ERL Theory in the COVID-19 Crisis

Self-regulated learning (SRL) vs. externally-regulated learning (ERL) Theory (de la Fuente, 2017) seeks to explain the combination of external and internal conditions that predispose adequate behavior in response to the pandemic or other situations. In summary, it proposes the following:

1. The *competence level* of *persons* is classifiable into *three levels of regulation* [1 = low (dysregulation, or lack of self-regulation); 2 = medium (reactive regulation); and 3 = high (proactive self-regulation)]. Based on prior research, *level of self-regulation,* as a personal characteristic, is a correlate of competence and of adequate management of different skills mentioned above, such as meta-motivation, meta-emotion and meta-behavior (de la Fuente et al., 2015, 2017). Consequently, it would be a good indicator of adequate self-care management in the COVID-19 crisis. The values 1, 2, and 3 indicate a range, from a higher level of personal regulation (3), i.e., the most proactive self-regulation; to a medium or nonregulatory level (2), not proactive in self-regulation; to the lowest level of self-regulation (1), which may even reflect dysregulation (pandemic-related examples: parties to see if the virus exists, risk contests, etc.).
2. *Interpersonal contexts* are equally classifiable into *three levels of external regulation* [1 = low (dysregulating context); 2 = medium (nonregulating context); and 3 = high (highly regulatory context)]. The *level of external regulation,* as a characteristic of the context, refers to how the context encourages adequate use of the different oversight competencies mentioned above, such as meta-motivation, meta-emotion and meta-behavior (de la Fuente et al., 2019b, 2020a). Consequently, it would be a good indicator of adequate contextual aspects in the COVID-19 crisis. The values 1, 2, and 3 indicate a range, from a context that more strongly encourages personal regulation (3), i.e., most actively promoting self-regulation; to a medium or nonregulatory level (2), the context does not offer external support for regulation; to the lowest level of external regulation (1), which may even reflect external dysregulation (pandemic-related examples: contradictory rules depending on the location, public figures who model noncompliance with the restrictions, etc.).
3. The *combination* of these two factors allows us to calculate an *interactive regulation index,* between 1 and 3, based on an average of the two regulation types, producing five possible combinations (de la Fuente et al., 2019b, 2020a). The proposed five-combination heuristic enables us to analyze the most common interactive scenarios in regulation of health behaviors during the pandemic. A *regulation average* of the person-context interaction is calculated as the mean of the individual’s regulation level and the regulatory level of the external context. For example, if a person has a low level of self-regulation (1 point), and their context offers a medium level of regulation (2 points), the resulting regulation average will be 1.5 points (2 + 1 = 3/2 = 1.5 point average); similarly, if the person has a medium level of regulation (2 points) and the context is low in regulation (1 point), it would produce the same average (2 + 1 = 3/2 = 1.5 point average). In another case, if a person has a high level of regulation (3 points) and interacts with a context that is low in regulation (1 point), the regulation average will be 2 points (3 + 1 = 4/2 = 2 points). The *regulation range* describing the person-context interaction increases from least favorable to most favorable: from 1 point (1-point personal Five-combination heuristicself-regulation in combination with 1-point external regulation), to a maximum of 3 points (3-point self-regulation in combination with 3-point external regulation). This heuristic, then, orders the possible combinations according to their regulation average, assigning them a *regulation rank* (regulation average of 1 = rank 1; regulation average of 1.5 = rank 2; regulation average of 2 = rank 3; regulation average of 2.5 = rank 4; and regulation average of 3 = rank 5). See Table 1.

**Table 1:** The Utility Model^TM^ hypothesized by self-regulated learning (SRL) vs. externally-regulated learning (ERL) theory (de la Fuente, 2017).

Combination level		Regulation aver/rank	Regulation trend	Control of behavior^*^		Epidemy control^*^	Health SR^*^
SR level (range) Personal	ER level (range) Contextual			High	Low		
**3** (3.85–5.00) **H**	**3** (2.84–5.00) **H**	3.0/**5**	**High-High**: high-regulation	++	−	*H*	*H*
**2** (3.10–3.84) **M**	**3** (2.84–5.00) **H**	2.5/**4**	**Medium-High**: regulation	+	−	*M-H*	*M-H*
**3** (3.85–5.00) **H**	**2** (2.35–2.83) **M**	2.5/**4**	**High-Medium**: regulation	+	−	*M-H*	*M-H*
**2** (3.10–3.84) **M**	**2** (2.35–2.83) **M**	2.0/**3**	**Medium**: non-regulation	=	=	*M*	*M*
**2** (3.10–3.84) **M**	**1** (1.00–2.34) **L**	1.5/**2**	**Medium-Low**: dys-regulation	−	+	*M-L*	*M-L*
**1** (1.00–3.09) **L**	**2** (2.35–2.83) **M**	1.5/**2**	**Low-Medium**: dys-regulation	−	+	*M-L*	*M-L*
**1** (1.00–3.09) **L**	**1** (1.00–2.34) **L**	1.0/**1**	**Low-Low**: high dys-regulation	−	++	*L*	*L*

* Effects analyzed in this investigation. Please see and analyze the differences with previous research reports (de la Fuente et al., 2019b, p. 12; de la Fuente et al., 2020a, p. 5).

SR level, personal level of self-regulation (1–3 range); ER level, contextual level of external regulation (1–3 range); H, high level; M, medium level; L, low level; ++, greater amount; −, lesser amount.

### Examples of SRL and ERL Levels in a Psychoeducational Analysis

#### Regarding Characteristics of the Self-Regulation Level (Internally Induced Regulation)

According to the SRL vs. ERL theoretical model (de la Fuente, 2017), persons will learn or become competent – to a greater or lesser degree – by having induced or predisposed themselves to different levels of self-regulation:

##### High Level of Regulation (Self-Regulation or Positive Proactivity Behavior)

Well-informed, accurate knowledge of the COVID-19 phenomenon. Actively seeks current, reliable information. Conceptual structure of facts, and current conceptual models: explanatory and functional models, maps of COVID-19 spread, hygiene measures, use of masks, current legislation, etc.
Well-established personal principles (ethical and/or moral), applied to the situation: coherency, common good, respect for rules, support for healthcare workers, etc.
Well-established skills in problem solving, negotiation, emotion management (Senay et al., 2010).
High level of meta-skills (self-regulation), and consequently, frustration management and adequate habits (Puchalska-Wasyl, 2014).
Inhibition of maladjusted behaviors leading to prohibited conduct or harm to the community.
High regulation of habits and attitudes. Enjoys complying with rules and takes it as an activity for self-improvement (resilience). Does not commit prohibited behaviors, even though they may be tempting in the short term.
High sensitivity and an attitude of co-responsibility and social commitment.
Commitment to limiting one’s behavior, perceiving it as a contribution to the common good. Does not have desires to do the opposite, absence of psychological reactance. See theory of psychological reactance in a later section (Brehm, 1966; Brehm and Brehm, 1981).

##### Medium Level of Self-Regulation (Non-Regulation or Reactivity Behavior)

Inconsistent, more limited knowledge of the COVID-19 phenomenon. Incomplete information of the phenomenon, with a conceptual structure that is inconsistent in some aspects: explanatory and functional models, maps of COVID-19 spread, hygiene measures, use of masks, current legislation, etc.
Personal principles (ethical and/or moral) are not well established and are inconsistently applied in the situation: coherency, common good, respect for rules, support for healthcare workers, etc.
Medium skills in problem solving, negotiation, and emotion management.
Medium level of meta-skills (self-regulation), and consequently, of frustration management and adequate habits.
Inconsistent inhibition of maladjusted behaviors that lead to prohibited conduct or harm to the community.
Medium regulation of habits and attitudes. Irregular compliance with rules (off and on), does not always accept them as an activity for personal improvement (resilience). Occasionally carries out prohibited behaviors.
Medium level of sensitivity and attitude of co-responsibility and social commitment.
Partial commitment to limiting one’s behavior, medium presence of psychological reactance (Brehm, 1966; Brehm and Brehm, 1981).

##### Low Level of Self-Regulation (Absence of Regulation or Self-Induced Dysregulation; Negative Proactivity Behavior)

Little knowledge, or distorted knowledge, of the COVID-19 phenomenon. Explanatory models and information are not up to date.
Nonexistent or distorted personal (ethical and/or moral) principles: “I have the right to.”
Little skill in problem solving, negotiation, and emotion management.
Low self-regulation, and consequently, low frustration management.
Pathological self-regulation, leading to prohibited conduct or harm to the community.
Poor regulation of habits and attitudes. Enjoys breaking rules and takes it as a reaffirmation of freedom. Attends prohibited parties, secretly violates legislation.
Low level of sensitivity and attitude of co-responsibility and social commitment.
Is bothered when behavior is limited, perceiving it as a threat to personal freedom, and has desires to do the opposite. This is the phenomenon of *psychological reactance*, a contrary, affective reaction in response to regulations or impositions that affect freedom and autonomy. This reaction is especially common when people feel forced to adopt a particular opinion or participate in a specific behavior that they do not want to do, thereby prompting behaviors aimed at restoring one’s autonomy (Brehm, 1966, 1972; Wicklund, 1974; Brehm and Brehm, 1981). For example, reactance often prompts people to adopt an opinion that is opposed to the beliefs or attitudes that they were encouraged or even coerced into adopting. As a consequence, reactance often increases resistance to persuasion (Brehm and Brehm, 1981). Reactance was proposed to explain many common examples of resistance in society, recognizing adverse effects of prohibition.

##### Causes of Reactance

Reactance is experienced whenever a free behavior is restricted (Brehm, 1966, 1972; Wicklund, 1974; Brehm and Brehm, 1981; Miller et al., 1993, 2006; Kelly and Nauta, 1997; Donnell et al., 2001; Naeem et al., 2020). A free behavior, in this context, is any act or choice that individuals might make now or very soon. Free behaviors that are perceived as especially important, that is, more important than other free behaviors, trigger observable reactance if they are frustrated. Moreover, when a broader range of free behaviors is restricted, reactance increases considerably. Research has shown that it declines with age and has an interaction effect with gender (Hong et al., 1994).

##### Consequences and Implications of Reactance

As the level of reactance rises, so does motivation to re-establish freedom. This response may appear even if people are not aware of this affective state. Reactance may evoke a number of *reactions:*

First, and perhaps most surprisingly, reactance may trigger *dysregulatory* behaviors that are opposed to the rules or courses of action that were imposed and encouraged (Buller et al., 1998; Seibel and Dowd, 2001). Specifically, boomerang effects are often seen, where individuals become *more* prone to perform the behavior that was restricted (Brehm, 1966). Alternatively, they may participate in similar but different acts than the behavior that has been restricted, such as smoking more often when drugs are banned; these are called related boomerang effects (see Quick and Stephenson, 2007a, 2008).

Second, other similar persons can also reestablish one’s sense of freedom. For example, consider an individual who cannot participate in a particular act, like smoking a cigarette. A close friend who performs a similar behavior, like smoking marijuana, restores in part the sense of freedom, and decreases reactance. This is called indirect restoration (Brehm and Brehm, 1981) or indirect boomerang effects (see Quick and Stephenson, 2007a, 2008).

Third, reactance promotes *unfavorable attitudes* toward the prohibition or the imposed behavior (see Dillard and Shen, 2005 and Rains and Turner, 2007). For example, smoking bans might encourage attitudes contrary to these restrictions. Similarly, the message itself could be perceived as flawed or misdirected (for example, Quick and Stephenson, 2007b).

Finally, reactance provokes adverse attitudes toward the source of any restriction. In other words, the persons who prohibit certain free behaviors are considered unfavorable (Miller et al., 2007).

#### Regarding Characteristics of External Regulation Levels (Regulation That Is Encouraged Externally)

According to the SRL vs. ERL theoretical model (de la Fuente, 2017), contexts can be educational --in greater or lesser degree-- by inducing or predisposing different levels of self-regulation.

##### High Level of External Regulation: Encouraging Individuals’ Regulation Externally

Defined as a situational educational context (family, schools, community context, and media, etc.) that promotes and encourages individuals’ behavioral self-regulation, as well as treatment adherence, in the desired behavioral direction. This involves the use of different strategies for inducing behavioral changes:

1. *Behavioral antecedents* design: clear and precise norms that lead to consequences; creating expectations of success and social desirability; and presenting adequate models: modeling from significant adults and peers.
2. Training users in *necessary competencies* [see the section on levels of competencies: knowing (precise information and adequate principles) + know-how (training in skills and meta-skills) + mindset (adequate motivation, through expectancy-value models)].
3. *Behavioral consequences* design: application of contingencies with high probability: intermittent reinforcement design and cost of response programs with well-designed contingency rates. Vicarious social reinforcement in adequate doses.

##### Medium Level of External Regulation: Neither Favoring nor Discouraging Individuals’ Regulation

Defined as a situational context (family, schools, community context, and media, etc.) that does not consistently promote or aid individuals’ behavioral self-regulation and treatment adherence (desired behavioral change). This involves the use of different strategies for noncontingent inducement of behavioral changes:

1. *Behavioral antecedents* design: norms are clear and precise, but do not lead to consequences; expectations of success or social desirability are not created; subjects are requested to make changes; and models presented are uncertain or ambiguous.
2. Partial or incomplete training in *necessary competencies* (see the section defining level of competencies): knowing + know-how + mindset are up to the subject, and depend on the subject’s level of prior competence.
3. *Behavior consequences* design: contingencies applied with low or uncertain probability, reinforcement and cost of response programs are poorly designed and with nonexistent external contingency rates (Mazar and Zhong, 2010; Longoni et al., 2014). Reinforcement is left up to the subject.

##### Low Level of External Regulation: Encouraging Individuals’ Dysregulation

Defined as a situational context (family, schools, community context, and media, etc.) that promotes or leads to individuals’ dysregulation and behaviors contrary to those intended; adherence to behavioral change contrary to the desired behavior. Different conditions are involved:

1. *Behavioral antecedents* design: infringement of norms leads to reinforcing consequences; inadequate expectations of success and social desirability are created; and harmful models are presented as adequate. Exposure to dysregulatory modeling.
2. Users inadequately trained in *necessary competencies* [see the section defining level of competencies: knowing (imprecise information and inadequate principles) + know-how (dysregulatory skills and meta-skills) + mindset (inadequate motivation, through expectancy-value models)].
3. *Behavioral consequences* design: contingencies applied with high probability to inadequate behaviors; intermittent reinforcement design and cost of response programs with irregular contingency rates.

## Implications For Psychoeducational Behavioral Intervention During the Covid-19 Crisis

### In the Sphere of Personal Competence

The design and development of programs to build competence in coping with the COVID-19 pandemic cannot be delayed. These should be based on the competencies model, assisting with training at each of its psychoeducational levels (Gagne, 1997), just as in its application to other problem areas.

Implementation of these preventive programs at all educational levels is essential, especially in reference to psychological or behavioral variables that predict self-regulation and adherence to proposed health treatment. We need educators that are well trained in this model, for proactive prevention of COVID-19 in schools at all levels.

### In the Educational Sphere of Family and School

#### Family and School Contexts That Externally Promote Regulation (Regulatory Contexts)

1. *Well-ordered behaviors and predictable environment.* In the family, the existence of flexible, reasonable norms that are understood by all promotes external regulation of sons and daughters. Environmental design is a first-order regulatory factor; as an antecedent of behavior, it helps to regulate it, since it constitutes a set of discriminatory signals that create expectations and behavioral value regarding what is expected to happen.
  The behaviors of adults and older siblings are essential elements of the regulatory environment, given that they act as daily behavior models and mold behavior with their reinforcing interactions, social punishment, withdrawals of attention, and response costs (Fuentes et al., 2015). In this regard, behavioral consequences should be regular, stable and consistent, and should be applied in such a way as to minimize psychological reactance, referred to earlier.
2. *Adequate, emotionally close regulation models.* Blunt prohibitions induce reactance and have no place in this context – except as a last resort of external control. Rules and consequences should be established and applied in an agreed-on manner, and above all, should be explained (this is what really educates and produces competence). It is necessary to express the principles, reasons, values, meaning and importance of the behavioral guideline that we are applying, and to have dialogue about them with our students or children. The effort involved, and even sacrifice, that many self-regulation behaviors involve, should also be discussed. It is essential that there is a relationship of affection and trust. This is what gives us influence and power of persuasion in our advice.
3. *Regulatory habits.* Self-regulation takes shape in one’s habits. This meta-skill requires practice in order to be established as a habit. Good habits, in turn, reinforce this meta-skill, by allowing it to be continuously in use.

### Family and School Contexts That Do Not Contribute Toward Regulation (Non-regulatory External Contexts)

1. *Ambivalent family context.* Characterized by an environment lacking in guidelines, permissive, with unspecified limits or rules, and few control contingencies that offer external regulation. Consequently, this environment has been called *laisser-faire* (Fuentes et al., 2015). It is assumed that each member of the family must self-regulate, and therefore, external help from outward signals or rules is not needed. All the weight falls on the individual member’s own self-regulation.
2. *Learning by trial and error is reinforced.* Behavioral initiative and learning by experience, discovery, and trial and error behaviors, are explicitly acknowledged and reinforced, meaning that behavioral excesses and deficits are accepted de facto. In the case of COVID-19, for example, parents will not remind or promote self-protection rules (mask use) or health rules (not attending mass events), but young people must make their own decisions whether to do so or not, according to their own level of self-regulation and competence regarding the problem.

Broken home environments, where parents are separated or absent, often tend toward this environmental design. Sometimes it is interpreted as a compromise, in order to alleviate and avoid conflict between parental rules that contradict each other. This encourages behavioral excesses and deficits, especially at ages where external regulation is necessary, such as adolescence and youth.

### Family and School Contexts That Promote Dysregulation (Dysregulatory External Contexts)

1. *Modeling behavioral excesses and deficits as a dysregulating factor.* When people are exposed to behavioral models of excesses or deficits, and such inadequate models are vicariously reinforced by the context, this increases their probability of being reproduced (Bandura, 1971). It is thus highly likely that inadequate habits and behaviors become established. Let us imagine a family where, during lockdown, the parents’ cope inadequately with the anxiety that this situation causes them by behavioral excess (excessive drinking, eating, and TV watching) or behavioral deficit (daily walking is discontinued and regular work hours are not kept). Through modeling and molding (intermittent social reinforcement), such behaviors promote coping strategies in their children that are also based on excesses or deficits, and not on self-regulation. In the case of families with divorced parents who have opposing childraising criteria, this explicitly contradictory model gives way to an alternation of contingencies applied by the two parents with their children.
  Another situation that induces dysregulation, for example, is when a child goes out without a mask and the parent applies social reinforcement, using dysregulatory social comments and attention reinforcement: “That’s the way, son, this mask stuff is for fags,” “let *them* be the ones to wear them,” etc.
2. *Excessive control as a dysregulating factor*. Researchers have consistently found results showing that high levels of parental psychological control predict high levels of internalizing and externalizing problems in adolescents (Barber et al., 1994, 2005, 2012; Barber, 1996). One study longitudinally examined two potential mediators (negative emotional reactions and psychological reactance) of the link between parental psychological control and internalizing outcomes (i.e., depressive symptoms) and externalizing outcomes (i.e., antisocial behavior; Laird and Frazer, 2020). Psychological control seems to involve two concurrent processes: (a) the observable behaviors exhibited by the parents (e.g., telling the child how to think), and (b) the emotional (e.g., feeling controlled or intrusive control). Psychological, emotional and behavioral reactions, and internalizing /externalizing behavior may represent stable patterns similar to traits and motivational responses (e.g., compliance or resistance) exhibited by the adolescent. Two potential reactions to experiencing psychological control are of particular interest:
  The first involves *feeling frustrated*, controlled, or invaded by the parent’s behavior. This construct has been labeled in previous work as negative emotional reaction and is similar to the need for frustration as operationalized by adolescents experience high levels of behavioral and psychological control as excess control. The adolescent is likely to feel frustrated, controlled or invaded by parental behavior and these emotional reactions can be expressed through internalizing or externalizing symptoms.
  The second, *psychological reactance* (Brehm, 1966), is defined as a “motivational state that is supposed to occur when a freedom is eliminated or threatened with elimination” (Brehm and Brehm, 1981, p. 37). Reactance is an unpleasant arousal in response to a threat or loss of freedom, motivating individuals to act to restore autonomy (Seltzer, 1983; Crawford et al., 2002; Steindl et al., 2015). Psychological control includes both external and internal threats that can produce psychological reactance. Psychological control poses an external threat by restricting a child’s behavior. And when the need for relatedness conflicts with the need for autonomy, there is an internal threat, because the child is forced to prioritize the satisfaction of one need over the other. Fulfilling the psychologically controlling parent satisfies the need for kinship while not meeting the need for autonomy, but resisting the psychological controlling parent may satisfy the child’s needs for autonomy at the expense of the child’s need for kinship. Reactance can produce internalizing symptoms when the child responds to internal threats, or externalizing symptoms when the child responds to external threats.

### In the Socio-Community Educational Sphere

#### Externally Regulatory Context

1. *Clear, precise rules have been adopted by the community (antecedent variable) as an external regulatory factor.* All citizens have been informed of the rules, which are clear, unambiguous and promote self-regulation. They are based on rational arguments, with true, objective information. They are adopted and internalized by the population: they are necessary, they create expectations of control, and have value because they are appropriate to the context (Silvia, 2005). Rules lead to positive and negative consequences (and there is high perceived likelihood of contingency).
2. *Correct, preventive behavioral modeling (antecedent variable) as an external regulatory factor.* In this context, adequate behaviors to be followed are shown publicly and modeled using psychologically meaningful models for the different ages and types of persons. In other words, external regulatory signals are designed that promote personal self-regulation. These models create success expectations and values of attainment that result in adequate behavior and commitment to prevention (expectancy-value model; Pekrun, 2007). Dysregulatory behaviors are not publicized or modeled, and news that predisposes dysregulatory behaviors is not broadcast.
3. *Competent citizens (educational level of competence variable).* In this context, the other citizens that one observes have an essential role; since they are competent, they become regulatory agents and promote regulation in their peers. We must not forget that learning by social modeling is especially effective among age peers, who have influence and impact value as significant models of identification (see the Social Learning Theory; Bandura, 1971). In other words, educated, competent persons become – in themselves – agents of external regulation for others, through social modeling. The group of friends and peers at each age level would be essential for this effect, for example, among adolescent citizens, young university students, and adults, especially when they perceive themselves to be non-risk groups.
4. *Recognized, reasonable contingencies have been adopted by the community (consequent variables).* In this type of context, the citizens share the contingencies and participate in their application. Consequently, there is a high probability that the foreseen consequences or contingencies are applied; citizens are involved and exercise hetero-regulation --and if applicable, behavioral hetero-control. In communities or cultures with a high sense of social discipline and shared co-responsibility, it is easier to create an effective regulatory environment design (Veling et al., 2008; Camacho et al., 2020).

### Non-regulatory Context (No Externally Induced Regulation)

1. No clear, precise rules that have been adopted by the community (antecedent variable) as an external regulatory factor, or rules are ambiguous. People are told to apply restrictions with ambiguous criteria or depend on their own criteria. The SRL vs. ERL theory predicts that this context requires more regulatory effort on the part of citizens. For example, if a person goes out and finds people who wear masks and people who do not, that person must decide whether to wear one based on their own criteria. The effort is even greater if there is no expectation of external contingencies from wearing or not wearing a mask.
2. Preventive behavioral modeling (antecedent variable), as an external regulatory factor, is confusing. Given that some people follow the rules and others do not, it is up to one’s own criteria to follow the behavior of certain models or others. If a person possesses psychological factors and personal characteristics that predispose them to risk, they are more likely to make an inadequate choice from the preventive point of view.
3. Level of competence in citizens (educational level of competence variable), is medium or low. The educational level or level of training for an emergency or pandemic of these characteristics is low or medium. This means that little knowledge of the facts, principles, skills, meta-skills, attitudes, values and habits needed for coping with this health emergency situation. Incompetent persons become inadequate elements of external regulation for others, by their modeling and social molding (Bandura, 1971).
4. No recognized, reasonable contingencies have been adopted by the community (consequent variables), nor are they perceived as likely. In this type of context, citizens perceive an absence of external contingencies, and they do not have the training to self-apply contingencies in a self-regulating way. In this case, the laisser-faire model prevails at the community level. Behavior compliance or noncompliance is left to the criteria of personal self-regulation. This means greater personal regulatory effort, given that the external context is not ordered in a regulatory way.

### Dysregulatory Context (Externally Induced Dysregulation)

1. *Clear, precise rules that encourage dysregulation (antecedent variable) become a dysregulating factor.* Creation of expectations and values (motivation) is toward transgression, or contrary to prevention rules, in certain more manipulable groups characterized by reactance or psychological needs (adolescents, young people, or adults without health competence). This can be observed in news reports of parties, crowds and big events without the use of masks, etc. The most extreme case of external dysregulation can be illustrated by the authorities expressly encouraging people to not wear masks as a sign of “personal freedom” or to attend dangerous events to show their commitment to a “noble cause.”
2. *Dysregulatory behavioral modeling is very explicit and powerful (antecedent variable).* Social learning theory clearly explains that social pressure can effectively achieve changes in one’s own criteria, even to an opposite direction. This is especially important in human developmental periods when peer pressure is greater. If a young person’s friends all send him or her messages to go to a mask-free party for fun, the chances increase that the young person will end up carrying out this dysregulatory behavior.
3. *Incompetent citizens (educational level of competence variable).* The educational level or level of training for an emergency or pandemic of these characteristics is very low, making the population more manipulable when presented with possible dysregulatory messages from their leaders. The absence of objective information, consolidated principles or personal strengths, lack of self-regulation, risk habits and maladjusted values make dysregulatory behavior very likely.
4. *The immediate contingencies that the subject receives are very clear and in favor of dysregulation (consequent variables).* If I do not go to the mask-free fun party, or do not go to the bar with my friends to drink and have a good time, I may face two undesired consequences: (1) social punishment from peers, with degrading comments on social networks or bullying within the group and (2) loss of the reinforcement and fun of the situation itself. Consequently, a high level of general competence and particularly of self-regulation is needed to be able to face these powerful immediate contingencies of a dysregulating nature.
5. *Psychological reactance of the person.* Previus research have reported that the threats of the COVID-19 pandemic and the subsequent restrictions to people’s freedom, social interaction, and closing of workplaces and shops have provoked public psychological reactance. Others research develops and demonstrates a conceptual framework that corroborates two bipolar behavioral results from psychological reactance in consumers: freedom of choice, satisfaction and resistance to persuasion. Anticipation of worry and confidence in the government positively moderated these relations.

Research indicates that certain language characteristics seem to evoke the perception that free behavior may be limited, provoking psychological reactance. In particular, language that is dogmatic, sometimes called *controlling* (Miller et al., 2007) or *explicit* (Grandpre et al., 2003), provokes reactance. As an illustration, Quick and Stephenson (2008) showed that *dogmatic messages* were perceived as more threatening, which provoked reactance, anger and unfavorable thoughts. Dogmatic messages include the following: imperatives (like “must” and “need”), absolute claims (“it cannot be denied that…” or “This problem is extremely serious”), disregard toward other perspectives (“Any reasonable person would agree that…”), and threatening warnings instead of impartial, objective information (Bushman, 1998). By contrast, messages that are less dogmatic do not provoke this sequence of reactions. Such messages are more likely to include the following: allusions to choice, like “You have the opportunity to…” or “It is up to you to choose…”; qualified statements, like “There is certain evidence that…” or “This problem is rather serious”; and objective and impartial information (Bushman, 1998). They avoid imperatives and derisory language. Many other studies have also confirmed that dogmatic language can promote reactance. For example, several studies have demonstrated that dogmatic language intended to reduce the consumption of alcohol provoked reactance (for example, Rains and Turner, 2007). Quick and Stephenson (2008) also showed that vivid language, where perceptual characteristics are described graphically, can also provoke a sensation of threat, and consequently, psychological reactance. Furthermore, for people who report high levels of reactance to *sensation-seeking traits*, dogmatic language was especially prone to promote reactance in the case of a vivid rather than pallid message.

*Individual differences.* Recent studies indicate that sensitivity to reactance varies from one individual to another. Dowd et al. (1994), for example, showed that persons who embrace autonomy and exhibit denial, dominance, independence, and mistrust are more inclined to experience psychological reactance. These findings are in alignment with Burgoon et al. (2002), who claimed that reactance would be more generalized in individuals who seek autonomy and feel that they are sufficiently competent and informed to choose their own course of action. People who are not especially sensitive to infringement on their freedom are more receptive to more directive or dogmatic persons. For example, Karno and Longabaugh (2005) carried out a study on clients who seek support for correcting their alcoholism. Clients who exhibited trait reactance were less prone to reduce their alcohol use when their counselors approached them with directives, initiated topics, communicated facts, interpreted comments, addressed resistance and formulated the main questions, instead of less directive approaches. However, clients that showed low levels of trait reactance changed their behavior even when the counselors were directive.

*Physical space.* Besides verbal communication, physical characteristics of an environment can also provoke a form of reactance, as Levav and Zhu (2009) uphold. For example, when people feel that their personal space is limited, they seem to demonstrate certain manifestations of reactance. In particular, they tend to select little-known or unusual products, demonstrating their independence through such selections. This search for independence may be prompted by reactance. When *physical space is limited,* individuals tend to feel stifled. As a consequence, they seek independence, restoring freedom by seeking variety.

## Conclusion

### The Covid-19 Behavioral Emergency in the Spheres of Health, Medicine-Biology and Educational Psychology

As the WHO claims, COVID-19 is an emergency in the health and medical-biological spheres. While the virus spreads at a fast rate, we have yet to fully understand its configuration, functionality, and structure. We are only in the early stages of learning how to provide medical care at all levels, whether preventive (vaccines) or treatment (pharmacological, ventilators, etc.). Worldwide growth of the pandemic continues to be steep.

Unlike certain classic diseases, in this case, the individual can play a more active role in controlling the development of COVID-19. Psychological and social/behavioral aspects are clearly central in its development and proliferation. For example, research has identified the individual’s self-regulation as a critical variable in a person’s self-care and social habits, as well as treatment adherence. Thus, a person’s behaviors in response to the crisis may be classified as regulatory (adequate), nonregulatory (doing nothing), or dysregulatory (doing the opposite of what is recommended). Another psychological variable of proven importance is coping strategies, used for managing anxiety and the stress provoked by situations with many unknowns. While these variables pertain to the individual, the context may also play an important role to encourage or discourage their presence.

For this reason, the *psychoeducational context* may also be regulatory, nonregulatory or dysregulatory. A regulatory external context would encourage individuals to self-regulate (appropriate messages and rules, social modeling, external monitoring, etc.). In the spread of COVID-19, this type of context predicts a more flattened curve of contagion, allowing the healthcare system the chance to respond. A nonregulatory context neither favors nor discourages self-regulation, it is left to the individual. Mathematical models of the unhindered spread of the epidemic point to a platykurtic curve that eventually brings the healthcare system to collapse. However, a dysregulatory context is also possible. This type of context encourages individual behavior that is directly opposed to what is appropriate. The epidemiological consequences of such behavior would be devastating, with contagion rapidly exploding (Zhu et al., 2020).

Psychology, as a behavioral science, is devoted to evaluating and intervening in such behavioral variables (psychoeducational and psychosocial). It is urgent that we recognize and address the psychosocial component of health-related issues, in addition to their medical-biological component. We must understand and act on behavioral, personal and contextual aspects, especially in critical situations such as the pandemic. An integrated, bio-psycho (educational)-social model (de la Fuente, 2020b) must underlie collaboration from the fields of Medicine, Biology and Psychology.

## Author Contributions

JF: design and initial writing. DK, MD, and YK: review and final writing. All authors contributed to the article and approved the submitted version.

### Conflict of Interest

The authors declare that the research was conducted in the absence of any commercial or financial relationships that could be construed as a potential conflict of interest.

## References

[ref1] Alemany-Arrebola I. Rojas G. Granda-Vera J. Custodio-Mingorance A. (2020). Influence of Covid-19 on the perception of academic self-efficacy, status anxiety and trait anxiety in college stydents. Front. Psychol. 11:570017. 10.3389/fpsyg.2020.570017, PMID: 33154727 PMC7586314

[ref2] Alessandri G. Filosa L. Tisak M. S. Crocetti E. Crea G. Avanzi L. (2020). Moral disengagement and generalized social trust as mediators and moderators of rule-respecting behaviors during the COVID-19 outbreak. Front. Psychol. 11:2102. 10.3389/fpsyg.2020.02102, PMID: 32973632 PMC7481453

[ref3] Arriaga P. Stevens F. Pavlova M. A. Gurreiro N. (2020). Coronavirus disease (COVID-19): the impact and role of mass media during the pandemic. Front. Psychol. 10.3389/fpsyg.2021.729238 PMC8419265 34497569

[ref4] Bandura A. (1971). Social learning theory. Morristown, N.J: General Learning Press.

[ref5] Barber B. K. (1996). Parental psychological control: revisiting a neglected construct. Child Dev. 67, 3296–3319. 10.2307/1131780, PMID: 9071782

[ref6] Barber B. K. Olsen J. E. Shagle S. C. (1994). Associations between parental psychological and behavioral control and youth internalized and externalized behaviors. Child Dev. 65, 1120–1136. 10.2307/1131309, PMID: 7956469

[ref7] Barber B. K. Stolz H. E. Olsen J. A. (2005). Parental support, psychological control, and behavioral control: assessing relevance across time, method, and culture. Monogr. Soc. Res. Child Dev. 70, 1–137. 10.1111/j.1540-5834.2005.00365.x, PMID: 16359423

[ref8] Barber B. K. Xia M. Olsen J. A. McNeely C. A. Bose K. (2012). Feeling disrespected by parents: refining the measurement and understanding of psychological control. J. Adolesc. 35, 273–287. 10.1016/j.adolescence.2011.10.010, PMID: 22177194

[ref9] Barzilay R. Moore T. M. Greenberg D. M. DiDomenico G. E. Brown L. A. White L. K.. (2020). Resilience, COVID-19-related stress, anxiety and depression during the pandemic in a large population enriched for healthcare providers. Transl. Psychiatry 10:291. 10.1038/s41398-020-00982-4 32820171 PMC7439246

[ref10] Brehm J. W. (1966). A theory of psychological reactance. New York: Academic Press.

[ref11] Brehm J. W. (1972). Responses to loss of freedom: A theory of psychological reactance. Morristown, NJ: General Learning Press.

[ref12] Brehm J. W. Brehm S. S. (1981). Psychological reactance: A theory of freedom and control. San Diego, CA: Academic Press.

[ref13] Brooks S. K. Webster R. K. Smith L. E. Woodland L. Wessely S. Greenberg N.. (2020). The psychological impact of quarantine and how to reduce it: rapid review of the evidence. Lancet 395, 912–920. 10.1016/S0140-6736(20)30460-8, PMID: 32112714 PMC7158942

[ref14] Buller D. B. Borland R. Burgoon M. (1998). Impact of behavioural intention on effectiveness of message features: evidence from the family sun safety project. Hum. Commun. Res. 24, 433–453. 10.1111/j.1468-2958.1998.tb00424.x

[ref15] Burgoon M. Alvaro E. Grandpre J. Voloudakis M. (2002). “Revisiting the theory of psychological reactance: communicating threats to attitudinal freedom” in The persuasion handbook: Developments in theory and practice. eds. Dillard J. P. Pfau M. (Thousand Oaks, CA: Sage), 213–232.

[ref16] Bushman B. J. (1998). Effects of warning and information labels on consumption of full-fat, reduced-fat, and no-fat products. J. Appl. Psychol. 83, 97–101. 10.1037/0021-9010.83.1.97, PMID: 9494441

[ref17] Cachón-Zagalaz J. Sánchez-Zafra M. Sanabrias-Moreno M. González-Valero G. Lara-Sánchez A. J. María Luisa Zagalaz-Sánchez M. L. (2020). Systematic review of the literature about the effects of the COVID-19 pandemic on the lives of school children. Front. Psychol. 11:569348. 10.3389/fpsyg.2020.569348, PMID: 33162910 PMC7591583

[ref18] Camacho A. C. L. F. Fuly P. D. S. C. dos Santos M. L. S. C. de Menezes H. F. (2020). Students in social vulnerability in distance education disciplines in times of COVID-19. Res. Soc. and Devel. 9:275973979. 10.33448/rsd-v9i7.3979

[ref19] Cao W. Fang Z. Hou G. Han M. Xu X. Dong J.. (2020). The psychological impact of the COVID-19 epidemic on college students in China. Psychiatry Res. 287:112934. 10.1016/j.psychres.2020.112934, PMID: 32229390 PMC7102633

[ref20] Castelnuovo G. De Giorgio A. Manzoni G. M. Treadway D. C. Mohiyeddini C. (2020). Psychological, behavioral, and interpersonal effects and clinical implications for health systems of the coronavirus (covid-19) pandemic: a call for research. Front. Psychol. 11:2146. 10.3389/fpsyg.2020.02146 33071842 PMC7541698

[ref21] Chacón-Fuertes F. Fernández-Hermida J. García-Vera M. P. (2020). La Psicología ante la Pandemia de la COVID-19 en España. La Respuesta de la Organización Colegial [psychology in the face of the COVID-19 pandemic in Spain. The collegiate Organization’s response]. Clínica y Salud 31, 119–123. 10.5093/clysa2020a18

[ref23] Crawford M. T. McConnell A. R. Lewis A. C. Sherman S. J. (2002). Reactance, compliance, and anticipated regret. J. Exp. Soc. Psychol. 38, 56–63. 10.1006/jesp.2001.1481

[ref24] de la Fuente J. (2015). *Competence for Studing, Learning and Performance ande Stress (CSLPS)*. Almería: Education & Psychology I+D+i.

[ref25] de la Fuente J. (2017). Theory of self- vs. externally-regulated LearningTM: fundamentals, evidence, and applicability. Front. Psychol. 8:1675. 10.3389/fpsyg.2017.01675, PMID: 29033872 PMC5627139

[ref26] de la Fuente J. (2020a). Medicine and psychology: From human hardware and software. Pamplona, Spain: University of Navarra.

[ref27] de la Fuente J. (2020b). The healthcare, medical-biological and psycho-social behavioral emergency of COVID-19. Available at: http://www.estres.investigacion-psicopedagogica.com/english/seccion.php?idseccion=16 (Accessed April 15, 2020).

[ref28] de la Fuente J. (2020c). A Structural equation model of protection and risk factors for university academic stress: analysis and implications for the COVID-19 emergency*. Front. Psychol.* 11 (in review). 10.3389/fpsyg.2021.562372 PMC8415087 34484015

[ref29] de la Fuente J. Amate J. González-Torres M. C. Artuch R. García-Torrecillas J. M. Fadda S. (2020a). Effects of levels of self-regulation and regulatory teaching on strategies for coping with academic stress in undergraduate students. Front. Psychol. 11:22. 10.3389/fpsyg.2020.00022 32082213 PMC7005059

[ref30] de la Fuente J. González-Torres M. C. Aznárez-Sanado M. Martínez-Vicente J. M. Peralta-Sánchez F. J. Vera M. M. (2019a). Implications of unconnected micro, molecular, and molar level research in psychology: the case of executive functions, self-regulation, and external regulation. Front. Psychol. 10:1919. 10.3389/fpsyg.2019.01919 31507487 PMC6719524

[ref31] de la Fuente J. Kauffman D. Dempsy M. Kauffman Y. (2020b). Coronavirus disease (COVID-19): psychoeducational variables involved in the health emergency. Front. Psychol. 10.3389/fpsyg.2022.961261 PMC9549808 36225683

[ref32] de la Fuente J. Kauffman D. Díaz-Orueta U. Kauffman Y. (2018). Adapting the research development and innovation (RD & I) value chain in psychology to the educational psychology area. Front. Psychol. 9:1188. 10.3389/fpsyg.2018.01188 30197609 PMC6117246

[ref33] de la Fuente J. Martínez-Vicente J. M. Peralta-Sánchez F. J. Garzón-Umerenkova A. Vera M. M. Paoloni P. (2019b). Applying the SRL vs. ERL theory to the knowledge of achievement emotions in undergraduate university students. Front. Psychol. 10:2070. 10.3389/fpsyg.2019.02070 31620044 PMC6760021

[ref34] de la Fuente J. Peralta-Sánchez F. J. Martínez-Vicente J. M. Sander P. Garzón-Umerenkova A. Zapata L. (2020c). Effects of self-regulation vs. external regulation on the factors and symptoms of academic stress in undergraduate students. Front. Psychol. 11:1773. 10.3389/fpsyg.2020.01773 32982819 PMC7480135

[ref35] de la Fuente J. Sander P. Martínez-Vicente J. M. Vera M. Garzón A. Fadda S. (2017). Combined effect of levels in personal self-regulation and regulatory teaching on meta-cognitive, on meta-motivational, and on academic achievement variables in undergraduate students. Front. Psychol. 8:232. 10.3389/fpsyg.2017.00232, PMID: 28280473 PMC5322205

[ref36] de la Fuente J. Zapata L. Martínez-Vicente J. M. Sander P. Cardelle-Elawar M. (2015). The role of personal self-regulation and regulatory teaching to predict motivational-affective variables, achievement, and satisfaction: a structural model. Front. Psychol. 6:399. 10.3389/fpsyg.2015.00399, PMID: 25964764 PMC4404977

[ref37] Dillard J. P. Shen L. (2005). On the nature of reactance and its role in persuasive health communication. Commun. Monogr. 72, 144–168. 10.1080/03637750500111815

[ref38] Donnell A. J. Thomas A. Buboltz W. C. (2001). Psychological reactance: factor structure and internal consistency of the questionnaire for the measurement of psychological reactance. J. Soc. Psychol. 141, 679–687. 10.1080/00224540109600581 11758045

[ref39] Dowd E. T. Wallbrown F. Sanders D. Yesenosky J. M. (1994). Psychological reactance and its relationship to normal personality variables. Cogn. Ther. Res. 18, 601–612. 10.1007/BF02355671

[ref40] Ebrahim S. H. Ahmed Q. A. Gozzer E. Schlagenhauf P. Memish Z. A. (2020). Covid-19 and community mitigation strategies in a pandemic. BMJ 368:m1066. 10.1136/bmj.m1066, PMID: 32184233

[ref41] Fuentes M. C. García F. Gracias E. Alarcón A. (2015). Los estilos parentales de socialización y el ajuste psicológico. Un estudio con adolescentes españoles [parental styles of socialization and psychological adjustment. A study with Spanish adolescents]. Revista de Psicodidáctica 20, 117–138. 10.1387/RevPsicodidact.10876

[ref42] Gagne R. M. (1997). Mastery learning and instructional design originally published in 1988, PIQ 1.1. Perform. Improv. Q. 10, 8–19.

[ref43] Gagne R. M. Briggs L. J. Wager W. W. (1988). Instructional design. New York: Rinehart and Winston Inc.

[ref44] Grandpre J. R. Alvaro E. M. Burgoon M. Miller C. H. Hall J. R. (2003). Adolescent reactance and anti-smoking campaigns: a theoretical approach. Health Commun. 15, 349–366. 10.1207/S15327027HC1503_6, PMID: 12788679

[ref45] Hagger M. S. Cameron L. D. Hamilton K. Hankonen N. Lintunen T. (eds.) (2020). “Changing behavior: a theory-and evidence-based approach” in The handbook of behavior change (England, London: Cambridge University Press), 1–14.

[ref46] Hong S. M. Giannakopoulos E. Laing D. Williams N. A. (1994). Psychological reactance: effects of age and gender. J. Soc. Psychol. 134, 223–228. 10.1080/00224545.1994.9711385, PMID: 8201818

[ref47] Huremovic D. (ed.) (2019). “Brief history of pandemics (pandemics throughout history)” in Psychiatry of pandemics. A mental health response to infection outbreak (New York: Springer), 7–35.

[ref48] Kanekar A. Sharma M. (2020). COVID-19 and mental well-being: guidance on the application of behavioral and positive well-being strategies. Healthcare 8:336. 10.3390/healthcare8030336, PMID: 32932613 PMC7551187

[ref49] Karno M. P. Longabaugh R. (2005). Less directiveness by therapists improves drinking outcomes of reactant clients in alcoholism treatment. J. Consult. Clin. Psychol. 73, 262–267. 10.1037/0022-006X.73.2.262, PMID: 15796633

[ref50] Kelly A. E. Nauta M. M. (1997). Reactance and thought suppression. Personal. Soc. Psychol. Bull. 23, 1123–1132.

[ref51] Kim H. S. Drolet A. (2003). Choice and self-expression: a cultural analysis of variety seeking. J. Pers. Soc. Psychol. 85, 373–382. 10.1037/0022-3514.85.2.373, PMID: 12916577

[ref52] Laird R. D. Frazer A. L. (2020). Psychological reactance and negative emotional reactions in the link between psychological control and adolescent adjustment. Soc. Dev. 29, 159–177. 10.1111/sode.12407

[ref53] Lazcano-Ponce E. Alpuche-Aranda C. (2020). Alfabetización en salud pública ante la emergencia de la pandemia por Covid-19 [public health literacy in the face of the Covid-19 pandemic emergency]. Salud Publica Mex. 62, 331–340. 10.21149/11408, PMID: 32304371

[ref54] Leung N. Chu D. Shiu E. Kwok-Hung C. McDevitt J. J. Hau B.. (2020). Respiratory virus shedding in exhaled breath and efficacy of face masks. Nat. Med. 26, 676–680. 10.1038/s41591-020-0843-2, PMID: 32371934 PMC8238571

[ref55] Levav J. Zhu R. (2009). Seeking freedom though variety. J. Consum. Res. 36, 600–610. 10.1086/599556

[ref56] Li W. Xie Y. (2020). The influence of family background on educational expectations: a comparative study. Chin. Sociol. Rev. 52, 1–26. 10.1080/21620555.2020.1738917

[ref57] Lippold J. V. Laske J. I. Hogeterp S. A. Duke É. Grünhage T. Reuter M. (2020). The role of personality, political attitudes and socio-demographic characteristics in explaining individual differences in fear of coronavirus: a comparison over time and across countries. Front. Psychol. 11:552305. 10.3389/fpsyg.2020.552305, PMID: 33071872 PMC7530433

[ref58] Liu J. J. Bao Y. Huang X. Shi J. Lu L. (2020). Mental health considerations for children quarantined because of COVID-19. Lancet Child Adolesc. Health. 4, 347–349. 10.1016/S2352-4642(20)30096-1, PMID: 32224303 PMC7118598

[ref59] Lohiniva A. L. Sane J. Sibenberg K. Puumalainen T. Salminen M. (2020). Understanding coronavirus disease (COVID-19) risk perceptions among the public to enhance risk communication efforts: a practical approach for outbreaks, Finland, February 2020. Euro Surveill. 25:2000317. 10.2807/1560-7917.ES.2020.25.13.2000317, PMID: 32265008 PMC7140598

[ref60] Longoni C. Gollwitzer P. M. Oettingen G. (2014). A green paradox: validating green choices has ironic effects on behavior, cognition, and perception. J. Exp. Soc. Psychol. 50, 158–165. 10.1016/j.jesp.2013.09.010

[ref61] Martarelli C. Pacozzi S. G. Bieleke M. Wolff W. (2020). High trait self-control and low boredom proneness help COVID-19 homeschoolers. *Front. Psychol.* 11 (in review). 10.3389/fpsyg.2021.594256 PMC7930236 33679514

[ref62] Martarelli C. Wolff W. (2020). Too bored to bother? Boredom as a potential threat to the efficacy of pandemic containment measures. Draff in review.

[ref63] Mazar N. Zhong C. (2010). Do green products make us better people? Psychol. Sci. 21, 494–498. 10.1177/0956797610363538, PMID: 20424089

[ref64] Miller W. R. Benefield R. G. Tonigan J. S. (1993). Enhancing motivation for change in problem drinking: a controlled comparison of two therapist styles. J. Consult. Clin. Psychol. 61, 455–461. 10.1037/0022-006X.61.3.455, PMID: 8326047

[ref65] Miller C. H. Burgoon M. Grandpre J. R. Alvaro E. M. (2006). Identifying principal risk factors for the initiation of adolescent smoking behaviors: the significance of psychological reactance. Health Commun. 19, 241–252. 10.1207/s15327027hc1903_6, PMID: 16719727

[ref66] Miller C. H. Lane L. T. Deatrick L. M. Young A. M. Potts K. A. (2007). Psychological reactance and promotional health messages: the effects of controlling language, lexical concreteness, and the restoration of freedom. Hum. Commun. Res. 33, 219–240. 10.1111/j.1468-2958.2007.00297.x

[ref67] Naeem M. A. Usman M. Ali M. Siddiqi U. I. (2020). COVID-19 restrictions and consumers’ psychological reactance toward offline shopping freedom restoration. Serv. Ind. J. 40, 891–913. 10.1080/02642069.2020.1790535

[ref68] Obrero-Gaitán E. Nieto-Escamez F. Zagalaz-Anula N. Cortés-Pérez I. (2020). An innovative approach for online neuroanatomy and neuropathology teaching based on 3D virtual anatomical models using leap motion controller. *Front. Psychol.* 11 (in review). 10.3389/fpsyg.2021.590196 PMC8273340 34262499

[ref69] Pekrun R. Frenzel A. C. Goetz T. Perry R. P. (2007). “The control-value theory of achievement emotions: an integrative approach to emotions in education” in Emotion in education. eds. Schutz P. A. Pekrun R. (London: Academic Press), 13–36.

[ref70] Proyecto Covid-19 (2020). Factores de difusión del COVId-19 en España [COVId-19 difusion factors in Spain]. *Instituto Carlos III*. Available at: https://portalcne.isciii.es/fdd/ (Accessed April 15, 2020).

[ref71] Puchalska-Wasyl M. M. (2014). When interrogative self-talk improves task performance: the role of answers to self-posed questions. Appl. Cogn. Psychol. 28, 374–381. 10.1002/acp.3007

[ref72] Quick B. L. Stephenson M. T. (2007a). Further evidence that psychological reactance can be modeled as a combination of anger and negative cognitions. Commun. Res. 34, 255–276. 10.1177/0093650207300427

[ref73] Quick B. L. Stephenson M. T. (2007b). The reactance restoration scale (RRS): a measure of direct and indirect restoration. Commun. Res. Rep. 24, 131–138.

[ref74] Quick B. L. Stephenson M. T. (2008). Examining the role of trait reactance and sensation seeking on perceived threat, state reactance, and reactance restoration. Hum. Commun. Res. 34, 448–476. 10.1111/j.1468-2958.2008.00328.x

[ref75] Rains S. A. Turner M. (2007). Psychological reactance and persuasive health communication: a test and extension of the intertwined model. Hum. Commun. Res. 33, 241–269. 10.1111/j.1468-2958.2007.00298.x

[ref76] Seibel C. A. Dowd E. T. (2001). Personality characteristics associated with psychological reactance. J. Clin. Psychol. 57, 963–969. 10.1002/jclp.1062, PMID: 11406807

[ref77] Seltzer L. F. (1983). Influencing the shape of resistance: an experimental exploration of psychological reactance and paradoxical directives. Basic Appl. Soc. Psychol. 4, 47–71.

[ref78] Senay I. Albarracin D. Noguchi K. (2010). Motivating goal-directed behavior through introspective self-talk: the role of the interrogative form of simple future tense. Psychol. Sci. 21, 499–504. 10.1177/0956797610364751, PMID: 20424090 PMC3626423

[ref79] Shen L. Dillard J. P. (2005). Psychometric properties of the Hong psychological reactance scale. J. Pers. Assess. 85, 74–81. 10.1207/s15327752jpa8501_07, PMID: 16083386

[ref80] Silvia P. J. (2005). Deflecting reactance: the role of similarity in increasing compliance and reducing resistance. Basic Appl. Soc. Psychol. 27, 227–284. 10.1207/s15324834basp2703_9

[ref81] Sokolowska J. Ayton P. Brandstätter E. (2020). Coronavirus disease (COVID-19): psychological reactions to the pandemic. Front. Psychol. 11:588910. 10.3389/fpsyg.2020.588910, PMID: 34526948 PMC8435768

[ref82] Spalleta G. Gianni W. Kivipelto M. Palmer K. (2020). Cognitive, psychological, and psychiatric consequences of the coronavirus (COVID-19) pandemic in the population of older persons with cognitive impairment, dementia, and/or neuropsychiatric disorders. Front. Psychol. 10.3389/fpsyt.2021.748963 PMC8551481 34721115

[ref97] Steindl C. Jonas E. Sittenthaler S. Traut-Mattausch E. Greenberg J. (2015). Understanding psychological reactance. Zeitschrift für Psychologie 223, 205–214. 10.1027/2151-2604/a000222 27453805 PMC4675534

[ref83] Taylor S. (ed.) (2019a). “Contemporary methods for managing pandemics” in The psychology of pandemics. Preparing for the next global outbreak of infectious disease (Newcastle upon Tyne, England: Cambridge Scholars Publishing), 15–23.

[ref84] Taylor S. (ed.) (2019b). “Psychological reactions to pandemics” in The psychology of pandemics. Preparing for the next global outbreak of infectious disease (Newcastle upon Tyne, England: Cambridge Scholars Publishing), 23–39.

[ref85] Taylor S. (ed.) (2019c). “Treating pandemic-related emotional distress” in The psychology of pandemics. Preparing for the next global outbreak of infectious disease (Newcastle upon Tyne, England: Cambridge Scholars Publishing, 99–107.

[ref86] Valadez M. D. López-Aymes G. Ruvalcaba N. A., Flores, Ortiz, E G.., Rodríguez C. J. Borges A. (2020). Emotions and reactions to the confinement by COVID-19 of children and adolescents with high abilities and community samples: a mixed exploratory study. Front. Psychol., 11:585587. 10.3389/fpsyg.2020.585587, PMID: 33329242 PMC7719706

[ref87] Veling H. Holland R. W. van Knippenberg A. (2008). When approach motivation and behavioral inhibition collide: behavior regulation through stimulus devaluation. J. Exp. Soc. Psychol. 44, 1013–1019. 10.1016/j.jesp.2008.03.004

[ref88] Westera W. (2001). Competences in education: a confusion of tongues. Competences in education: a confusion of tongues. J. Curric. Stud. 33, 75–88. 10.1080/00220270120625

[ref89] Whillans A. Mayer C. H. Wong P. P. T. (2020). COVID-19 and existential positive psychology (PP2.0): the new science of self-transcendence. Front. Psychol. 10.3389/fpsyg.2021.800308 PMC8699172 34956025

[ref90] WHO Regional Office for Europe (2020). Mental health and psychological resilience during the Covid-19 pandemic. Available at: http://www.euro.who.int/en/health-topics/health-emergencies/coronavirus-covid-19/news/news/2020/3/mental-health-and-psychological-resilience-during-the-covid-19-pandemic (Accessed April 15, 2020).

[ref91] Wicklund R. A. (1974). Freedom and reactance. New Jersey, USA: Lawrence Erlbaum.

[ref92] Wilke N. G. Howard A. H. Pop D. (2020). Data-informed recommendations for services providers working with vulnerable children and families during the COVID-19 pandemic. Child Abuse Negl. 110:104642. 10.1016/j.chiabu.2020.104642, PMID: 32753231 PMC7392096

[ref93] Wolff W. Martarelli C. Schüler J. Bieleke M. (2020). High boredom proneness and low trait self-control impair adherence to social distancing guidelines during the COVID-19 pandemic. Int. J. Environ. Res. Public Health 17:5420. 10.3390/ijerph17155420, PMID: 32731369 PMC7432636

[ref94] Yoshikawa H. Wuermli A. J. Britto P. R. Dreyer B. Leckman J. F. Lye S. J.. (2020). Effects of the global COVID-19 pandemic on early childhood development: short-and long-term risks and mitigating program and policy actions. J. Pediatr. 223, 188–193. 10.1016/j.jpeds.2020.05.020, PMID: 32439312 PMC7234941

[ref95] Zaccoletti S. Camacho A. Correia N. Aguiar C. Mason L. Alves R. A. (2020). Parents’ perceptions of student academic motivation during the COVID-19 lockdown: a cross-country comparison. *Front. Psychol.* 11 (in review). 10.3389/fpsyg.2020.592670 PMC7775314 33391114

[ref96] Zhu S. Wu Y. Zhu C. -Y. Hong W. -C. Yu Z. -X. Chen Z. -K.. (2020). The immediate mental health impacts of the COVID-19 pandemic among people with or without quarantine managements. Brain Behav. Immun. 87, 56–58. 10.1016/j.bbi.2020.04.045, PMID: 32315758 PMC7165285

